# Real-world andexanet alfa utilization and the association between delay in administration due to hospital transfer and all-cause inpatient mortality

**DOI:** 10.1016/j.rpth.2025.102688

**Published:** 2025-01-24

**Authors:** Huiqiao Fan, Youssef Bessada, Craig I. Coleman

**Affiliations:** Department of Pharmacy Practice, School of Pharmacy, University of Connecticut, Storrs, Connecticut, USA

**Keywords:** andexanet alfa, factor Xa inhibitors, factor Xa reversal, hemorrhage, reversal agents

## Abstract

**Background:**

Evaluations of andexanet alfa for the reversal of factor Xa inhibitor-associated bleeding have been small, with cohorts drawn from single/limited sites. Delays in providing anticoagulation reversal due to hospital transfer may result in poorer outcomes.

**Objectives:**

To describe the characteristics and outcomes of andexanet alfa users and evaluate the association between delay in andexanet alfa administration due to transfer from a different acute care hospital and the incidence of all-cause inpatient mortality.

**Methods:**

This was a retrospective study using National Inpatient Sample data. Hospitalizations with procedural codes for andexanet alfa and a billing code for bleeding were included. Descriptive analysis was performed, as was multivariable logistic regression, to estimate the odds ratio and 95% CI for the association between andexanet alfa delayed due to transfer from a different acute care hospital and all-cause inpatient mortality.

**Results:**

From 2019 to 2021, 4210 hospitalizations occurred in adults receiving andexanet alfa and a bleed. Most were hospitalized with intracranial hemorrhage (62.0%). The incidence of all-cause inpatient mortality was 16.6% (95% CI, 14.3%-19.3%), mean hospital stays lasted 9.1 days (95% CI, 8.4-9.8), and mean hospital costs were $73,600 (95% CI, $65,000-$82,200). Of all cases, 18.5% were transferred from a different acute care hospital prior to receiving andexanet alfa. Cases with hospital transfer had an 82% increased odds of all-cause inpatient mortality (95% CI, 17%-183%) but did not reach statistical significance when the population was limited to intracranial hemorrhage (odds ratio, 1.51; 95% CI, 0.88-2.60).

**Conclusion:**

Delay in administering andexanet alfa due to hospital transfer may be associated with increased all-cause mortality.

## Essentials

• Delaying anticoagulation reversal due to hospital transfer may result in poorer outcomes.
• This was a retrospective study using National Inpatient Sample data from 2019 to 2021.
• Of 4210 cases, 18.5% were transferred from a different hospital prior to andexanet alfa use.
• Hospital transfer was associated with an 82% (95% CI, 17%-183%) increased odds of inpatient death.

## 1 Introduction

Andexanet alfa received accelerated conditional approval from the United States (US) Food and Drug Administration in 2018 as the first specific reversal agent for apixaban- or rivaroxaban-associated life-threatening or uncontrolled bleeding [1]. The andexanet alfa, a Novel Antidote to the Anticoagulation Effects of FXa Inhibitors (ANNEXA) 4 trial, a single-arm trial with no comparator, showed that in patients with oral factor (F)Xa inhibitor- or enoxaparin-associated major bleeding, andexanet alfa rapidly and significantly reduced anti-FXa activity in apixaban- and rivaroxaban-treated patients by >90% and achieved excellent or good hemostatic efficacy at 12 hours in 80% of patients [2]. The randomized ANNEXA-I trial demonstrated that andexanet significantly improved hemostasis compared with usual care (85.5% prothrombin complex concentrate [PCC]; adjusted difference, 13.4%; 95% CI, 4.6%-22.2%), albeit at an increased risk of thrombosis (difference, 4.6%; 95% CI, 0.1%-9.2%) [3]. Evaluations of andexanet alfa patients have largely been smaller single-arm assessments with cohorts drawn from single or limited centers [4,5]. Thus, there remains a need for larger, nationally representative, real-world evaluations describing andexanet alfa use and outcomes. Moreover, studies have suggested that delay in anticoagulation reversal, either symptom-to-treatment time > 60 minutes or door-to-needle time > 30 minutes, can result in increased mortality and/or need for hospice care [[6], [7], [8]]. Whether other methods of measuring delay in andexanet alfa administration, such as delay due to hospital transfer, show a similar association is unclear.

We sought to perform a study in a large, nationally representative cohort of hospitalizations for bleeding in which andexanet alfa was used to describe patient demographics, bleeding characteristics, comorbidities, and outcomes, as well as to evaluate the association between delay in reversal of bleeding due to the need for transfer from a different acute care hospital and the incidence of all-cause inpatient mortality.

## 2 Methods

We performed a retrospective cohort study using 2019-2021 National Inpatient Sample (NIS) data. The NIS is a database comprised of hospital discharges, developed by the Healthcare Cost and Utilization Project (HCUP) and sponsored by the Agency for Healthcare Research and Quality. The NIS is a 20% stratified randomized sampling of all hospital discharges drawn from participating states and covers >97% of the US population of all payer types. HCUP databases conform to the definition of a limited data set. Under Health Insurance Portability and Accountability Act, review by an institutional review board is not required for use of limited data sets.

Inclusion criteria for this study consisted of hospitalizations in adults with an International Classification of Diseases 10th Revision (ICD-10) procedural code XW03372 or XW04372, indicating the administration of andexanet alfa, and an inpatient billing code indicating a bleed. ICD-10 procedural codes for andexanet alfa were the result of the New Technology Add-On Payment (NTAP) program, which incentivizes and provides incremental payment for medical or new technologies that would otherwise be inadequately paid for [9]. The data years 2019 to 2021 were chosen as they represent full years of NTAP program inclusion for andexanet alfa. Bleed codes were drawn from published ICD-10 schemas [[10], [11], [12]]. Cases were categorized into mutually exclusive categories, including intracranial hemorrhage (ICH), non-ICH critical organ bleeds (as defined by the International Society on Thrombosis and Haemostasis) [13], gastrointestinal bleeds, and other bleeds. If there was more than 1 bleed type, they were placed in the most severe category. Cases without a bleeding code from the predefined schema had their ICD-10 billing codes independently screened by 2 investigators (H.F., Y.B.) for codes indicating a bleeding complication. Cases were excluded if they did not have an ICD-10 code indicating a bleed.

Our first objective was to describe demographics, bleeding characteristics, comorbidities, and hospitalization-associated outcomes within andexanet alfa users. Outcomes of interest included all-cause inpatient mortality, hospital length of stay (LOS), and all-cause total hospital treatment costs. As NIS provides charge data, charges were converted to costs using HCUP-supplied cost-to-charge ratios and inflated to 2023 US$ using the Consumer Price Index for Medical Care [14]. The second objective of our study was to assess whether the need for patient transfer from a different acute care hospital to the one in which they received andexanet alfa was associated with an increased odds of all-cause inpatient mortality.

NIS data were compiled using a complex survey design, requiring the use of survey-specific analysis methods that simultaneously account for clustering and stratification and the potential for sampling bias. Doing so allows the weighting of hospital stays to generate nationally representative estimates with accompanying 95% CIs [15]. We performed descriptive analysis on the weighted set of included hospital stays and report frequencies and means. Multivariable logistic regression was performed to compare the odds with 95% CIs of experiencing all-cause inpatient mortality among stays in which patients were transferred or not transferred from a different acute care hospital after adjustment for baseline covariates. Covariates included age, sex (male or female), race, region of hospital location, payer, year of hospitalization, bleed type, traumatic nature of bleeds, presence of hypertension, atrial fibrillation, heart failure, ischemic heart disease, diabetes, chronic kidney disease, history of venous thromboembolism, history of stroke, obesity, and cancer. Upon review of a correlation matrix of the above covariates, no substantial (−.5 < correlation coefficient > .5) multicollinearity was detected. Further analysis was performed on a subset of patients restricted to ICH bleeds. Database management and statistical analysis were performed using IBM SPSS Statistics, version 28.0.1.1 (IBM, SPSS Inc).

## 3 Results and Discussion

From 2019 to 2021, there were 4585 weighted hospitalizations with ≥1 ICD-10 procedural code for the use of andexanet alfa. Of these, 4210 weighted hospitalizations occurred in an adult patient with an ICD-10 code documented bleed (Table 1). Most hospitalizations were for an ICH (62.0%), followed by gastrointestinal (18.3%), other (12.1%), and non-ICH critical organ bleeding (7.6%). Of all bleeds, 30.2% were traumatic in nature. The mean age was 75.0 years; 44.9% were women, and the majority (78.7%) were White. The most common indications for anticoagulation included atrial fibrillation (72.3%) and a history of venous thromboembolism (18.2%).

**Table 1:** Demographics and comorbidities of patient hospitalizations utilizing andexanet alfa.

Characteristics	Andexanet alfa % (95% CI)		
	All^b^ *N* = 4210	Transferred in^a^	
		Yes *N* = 780	No *N* = 3430
Demographics			
Age (y), mean (95% CI)	75.0 (74.1-75.8)	74.4 (72.3-76.4)	75.1 (74.2-76.0)
Sex			
Female	44.9 (41.3-48.5)	41.0 (33.6-48.9)	45.8 (41.7-49.9)
Race or ethnicity			
White	78.7 (75.5-81.6)	74.4 (67.7-80.0)	79.7 (76.1-82.9)
Other	21.3 (18.4-24.5)	25.6 (20.0-32.3)	20.3 (17.1-23.9)
Hospital region			
Northeast	27.3 (23.5-31.5)	30.8 (22.2-40.9)	26.5 (23.0-30.3)
Midwest	29.7 (26.0-33.7)	28.2 (20.6-37.3)	30.0 (25.9-34.5)
South	32.4 (28.6-36.5)	33.3 (25.6-42.1)	32.2 (27.9-36.8)
West	10.6 (8.6-12.9)	7.7 (3.9-14.8)	11.2 (9.0-13.9)
Primary payer			
Medicare	80.9 (77.9-83.6)	78.8 (71.8-84.5)	81.3 (78.0-84.3)
Medicaid	4.4 (3.2-6.0)	4.5 (2.2-8.8)	4.4 (3.1-6.1)
Commercial/private	13.2 (11.0-15.7)	14.1 (9.3-20.9)	13.0 (10.6-15.7)
Other	1.5 (0.9-2.7)	2.6 (1.0-6.6)	1.3 (0.7-2.5)
Year			
2019	22.1 (17.1-28.0)	19.9 (12.2-30.7)	22.6 (17.5-28.6)
2020	32.8 (26.8-39.3)	34.0 (24.2-45.3)	32.5 (26.5-39.2)
2021	45.1 (38.6-51.8)	46.2 (35.7-56.9)	44.9 (38.1-51.9)
Transferred to the hospital	18.5 (15.7-21.8)	--	--
Bleed type			
ICH	62.0 (58.2-65.6)	66.7 (58.0-74.3)	60.9 (57.0-64.7)
Non-ICH critical organ bleed	7.6 (6.0-9.5)	5.1 (2.6-10.0)	8.2 (6.4-10.4)
Gastrointestinal	18.3 (15.7-21.2)	16.0 (10.7-23.4)	18.8 (16.1-21.9)
Other bleeds	12.1 (10.0-14.6)	12.2 (7.8-18.5)	12.1 (9.7-15.0)
Traumatic bleed	30.2 (27.0-33.5)	32.7 (25.2-41.2)	29.6 (26.3-33.1)
Comorbidities			
Atrial fibrillation	72.3 (69.0-75.4)	73.7 (66.0-80.2)	72.0 (68.5-75.3)
Ischemic heart disease	34.7 (31.5-38.0)	30.8 (24.4-37.9)	35.6 (32.2-39.1)
Diabetes	32.1 (28.9-35.4)	31.4 (24.0-39.9)	32.2 (28.5-36.1)
Hypertension	81.5 (78.7-84.0)	82.1 (75.2-87.3)	81.3 (78.3-84.1)
History of stroke	14.3 (11.8-17.1)	9.6 (5.5-16.2)	15.3 (12.7-18.3)
History of venous thromboembolism	18.2 (15.7-20.9)	17.9 (12.7-24.7)	18.2 (15.5-21.2)
Obesity	13.8 (11.6-16.3)	14.7 (9.8-21.6)	13.6 (11.1-16.5)
Chronic kidney disease	29.3 (26.5-32.3)	30.1 (23.3-37.9)	29.2 (26.0-32.5)
Heart failure	36.6 (33.4-39.9)	37.8 (30.7-45.5)	36.3 (32.7-40.0)
Cancer	10.8 (8.9-13.0)	7.1 (4.2-11.7)	11.7 (9.5-14.2)

ICH, intracranial hemorrhage.

a Transferred in from a different acute care hospital.

b Given the retrospective nature of the data analysis, andexanet use or the presence of a comorbid disease diagnosis was made based on the presence or absence of billing codes. The absence of billing codes, suggesting andexanet use or comorbidity exists, was assumed to represent the absence of use/disease. There were no missing age values in the andexanet cohort.

Among all bleeding-related hospitalizations in which andexanet alfa was administered, the incidence of all-cause inpatient death was 16.6% (95% CI, 14.3%-19.3%). The mean hospital LOS was 9.1 days (95% CI, 8.4-9.8), and the mean all-cause hospital costs were $73.6 x 1000 (95% CI, $65.0-$82.2 x 1000; Table 2).

**Table 2:** Outcomes of hospitalizations utilizing andexanet alfa.

Outcomes	All^b^ *N* = 4210	Transferred in^a^	
		Yes *n* = 780	No *n* = 3430
Died in hospital, % (95% CI)	16.6 (14.3-19.3)	24.4 (18.4-31.5)	14.9 (12.4-17.8)
LOS (d), mean (95% CI)^c^	9.1 (8.4-9.8)	11.7 (9.2-14.2)	8.6 (7.8-9.3)
Hospital costs ($ × 1000), mean (95% CI)	73.6 (65.0-82.2)	83.4 (70.0-96.9)	71.3 (61.4-81.3)

LOS, length of stay.

a Transferred in from a different acute care hospital.

b There were no missing values in the andexanet cohort.

c LOS for the hospitalization following the transfer only.

Patients were transferred in from a different acute care hospital and received andexanet alfa 18.5% of the time (N = 780 hospitalizations). Following multivariable logistic regression, transfer from a different acute care hospital prior to receiving andexanet alfa was associated with an 82% increased odds of all-cause inpatient mortality (odds ratio [OR], 1.82; 95% CI, 1.17-2.83) vs not being transferred (Table 3). Other covariates found to be associated with an increased odds of all-cause inpatient mortality included ICH (crude incidence: 18.2% vs 8.8%; OR, 3.12; 95% CI, 1.48-6.54) and gastrointestinal bleed types (crude incidence: 20.1% vs 8.8%; OR, 2.55; 95% CI, 1.12-5.79 compared with other bleed types). Traumatic bleeds were associated with lower odds of all-cause inpatient mortality compared with spontaneous bleeds (crude incidence: 13.0% vs 18.2%; OR, 0.47; 95% CI, 0.28-0.79).

**Table 3:** Result of multivariable logistic regression.

Covariates	All-cause inpatient mortality OR (95% CI)
Transferred to the hospital	
No	Reference
Yes	1.82 (1.17-2.83)
Demographics	
Age, y (increase per year)	0.98 (0.96-1.00)
Sex	
Female	0.82 (0.56-1.19)
Male	Reference
Race	
White	0.79 (0.50-1.24)
Other	Reference
Hospital region	
Northeast	0.61 (0.33-1.15)
Midwest	0.68 (0.38-1.22)
South	0.60 (0.33-1.10)
West	Reference
Primary payer	
Medicare	0.99 (0.20-4.88)
Medicaid	1.66 (0.29-9.48)
Commercial/private	1.45 (0.28-7.42)
Other	Reference
Year	
2019	1.32 (0.78-2.23)
2020	0.69 (0.45-1.04)
2021	Reference
Bleed type	
ICH	3.12 (1.48-6.54)
Non-ICH critical organ bleed	0.89 (0.27-2.98)
Gastrointestinal	2.55 (1.12-5.79)
Other bleeds	Reference
Traumatic bleed	
No	Reference
Yes	0.47 (0.28-0.79)
**Comorbidities**	
Atrial fibrillation	
No	Reference
Yes	0.80 (0.47-1.34)
Ischemic heart disease	
No	Reference
Yes	0.76 (0.50-1.14)
Diabetes	
No	Reference
Yes	1.10 (0.75-1.61)
Hypertension	
No	Reference
Yes	0.75 (0.46-1.23)
History of stroke	
No	Reference
Yes	0.74 (0.40-1.38)
History of venous thromboembolism	
No	Reference
Yes	0.70 (0.39-1.26)
Obesity	
No	Reference
Yes	0.69 (0.37-1.27)
Chronic kidney disease	
No	Reference
Yes	1.04 (0.68-1.59)
Heart failure	
No	Reference
Yes	1.21 (0.79-1.85)
Cancer	
No	Reference
Yes	0.62 (0.30-1.29)

ICH, intracranial hemorrhage.

When we limited the inclusion of hospital stays to those for ICH (N = 2610 stays, 62.0%), of which 19.9% (520 stays) began with a transfer from a different acute care hospital, the odds of all-cause inpatient mortality associated with delay of reversal due to hospital transfer was no longer significant (OR, 1.51; 95% CI, 0.88-2.60). When limited to gastrointestinal bleeds only (N = 770 stays, 18.3%), of which 18.3% (141 stays) began with a transfer from a different acute care hospital, the odds of all-cause inpatient mortality associated with delay of reversal due to hospital transfer was (OR, 3.47; 95% CI, 1.21-9.93).

In the present study, we analyzed a large, nationally representative cohort to describe andexanet alfa user hospitalizations’ characteristics and outcomes. Compared with ANNEXA-4 [2], the phase 3b trial that provided evidence for conditional Food and Drug Administration approval of andexanet alfa, the types of patients receiving andexanet alfa in our study appeared generally consistent. The mean age in both studies was in the mid to late 70s, there was approximately a 1:1 male-to-female ratio, patients were mostly White, and predominantly had atrial fibrillation as an underlying indication for anticoagulation. About two-thirds of patients/hospitalizations in both studies had/were for an ICH, and nearly 20% were gastrointestinal bleeds. Despite these similarities, differences in all-cause mortality rates were observed, with our study showing 16.6% inpatient mortality (over a mean LOS of 9.1 days) compared with ANNEXA-4’s 8.2% incidence of mortality within the first 2 weeks after andexanet alfa administration. A prior meta-analysis of anticoagulation reversal agent studies reported a mortality rate of 13.4% (95% CI, 10.6%-16.1%) among studies <30 days in duration [3]. While it is difficult to compare mortality incidence between clinical trials and retrospective studies, differences in mortality rates observed may be attributed to our real-world cohort having bleeds that are more severe or less amenable to anticoagulation reversal. For example, in ANNEXA-4 [2], patients were excluded if they had an expected survival of <1 month, an ICH with Glasgow Coma Scale score < 7, or hematomas > 60 mL, whereas our study may have included patients outside these exclusion criteria. Mortality differences may also have been related to the hospital transfer itself.

We found that patients transferred in from a different acute care hospital prior to receiving andexanet alfa were associated with an 82% increased odds of inpatient mortality. Delays in the administration of reversal agents have been shown to be associated with poorer outcomes in anticoagulation-associated bleeding. Sheth et al. [6] evaluated the Get With The Guidelines-Stroke registry and found that anticoagulation-associated patients with ICH treated with reversal interventions were associated with a 22% (95% CI, 8%-38%) increased odds of inpatient death or hospice discharge if their door-to-treatment time was >60 minutes (vs 60 minutes or less). In a second study, Dobesh et al. [7] found that in a cohort including all bleed types, a door-to-start of andexanet alfa administration time of ≥30 minutes was associated with an increased odds of inpatient mortality (OR, 1.72; 95% CI, 0.94-3.40), although only reaching statistical significance when the analysis was limited to the ICH subgroup (OR, 2.46; 95% CI, 1.12-6.22) [8]. Our analysis showed an increased odds of all-cause inpatient mortality in the gastrointestinal cohort who had a delay in andexanet because of the hospital transfer but did not show a significantly increased risk of mortality for patients with ICH. The failure to show a significant difference in our ICH patient subset is likely a result of an underpowered analysis (based on a 40% reduced sample size). It should be noted that this was an exploratory analysis only; patients with ICH already have a poor prognosis despite any delay in reversal agent administration, and the OR for the ICH population was generally consistent with the overall OR, which was significant. Analysis of other bleeding subtypes could not be reported due to Agency for Healthcare Research and Quality/HCUP size restrictions. While the reason for transfer from another inpatient acute care hospital in our study was not known, it may be, at least in part, due to hospitals not having andexanet alfa available [16]. As a result of this recent evidence, a consensus statement published by Li et al. [17] called for the widespread integration of time-focused metrics (including time-to-reversal) to improve ICH management, a key pillar of which is effective and timely anticoagulation reversal. Delays in reversal due to hospital transfer may be a major limiting factor to the integration of this practice.

Interestingly, we also found that hospitalizations for both an ICH and gastrointestinal bleed were associated with similar and substantial incidences of inpatient mortality. While prior studies suggest ICH is associated with a higher mortality rate than extracranial hemorrhage, including gastrointestinal bleeds [2,4], gastrointestinal bleeds can also be associated with substantial mortality, particularly in the presence of hemodynamic instability or shock, advanced age, and/or multiple comorbidities [18,19]. The 20.1% inpatient mortality rate among gastrointestinal hospitalizations observed in our study suggests that the use of andexanet alfa for gastrointestinal bleeding was mainly reserved for the most severe of these bleed types.

There were several limitations to our study. First, NIS does not have medication use data. Therefore, andexanet alfa had to be identified through NTAP ICD-10 coding, which could result in missed cases [9]. In addition, we could not verify that andexanet alfa was used in accordance with the US label [1], rule out PCC use prior to transfer, or confirm oral FXa inhibitor use prior to andexanet alfa administration. Delay in PCC use may also be associated with increased mortality but could not be assessed as NTAP codes for its use to reverse bleeding are not available. Second, the use of transfer status from another acute care hospital may be a less precise proxy for the delay in andexanet alfa administration than what has been used previously. Prior studies [[6], [7], [8]] analyzed time delays (symptom-to-administration time or door-to-administration time) in minutes, which was a level of detail we could not obtain in the NIS data. Third, the reason for transfer to NIS is not available. Fourth, to address the risk of confounding bias, our analysis used multivariable logistic regression. However, despite the use of regression, residual confounding from unmeasured covariates (eg, hemodynamic characteristics) in nonrandomized studies can never be fully ruled out. Fifth, we were not able to evaluate out-of-hospital mortality nor identify thrombotic events that occurred during the bleeding admission due to a lack of data in the NIS and an absence of reliable coding algorithms, respectively.
