# Supplementary material for: Real-world andexanet alfa utilization and the association between delay in administration due to hospital transfer and all-cause inpatient mortality
Source: Res Pract Thromb Haemost. 2025 Jan 24;9(1):102688. doi: 10.1016/j.rpth.2025.102688 (PMC11889373; doi:10.1016/j.rpth.2025.102688)
Supplement: Supplemental Materials [file mmc1.docx]

**Supplemental Appendix**

**Supplemental Table 1. International Classification of Diseases-10^th^ Revision Billing Codes Used to Identify Bleeding Type and Comorbidities^1-3^**

ICD-10 = Internation Classification of Diseases – Tenth Revision; ICH = intracranial hemorrhage

| **Bleed Type** | **ICD-10 Codes** |
| --- | --- |
| Intracranial Hemorrhage | S064X0A, S064X0D, S064X0S, S064X1A, S064X1D, S064X1S, S064X2A, S064X2D, S064X2S, S064X3A, S064X3D, S064X3S, S064X4A, S064X4D, S064X4S, S064X5A, S064X5D, S064X5S, S064X6A, S064X6D, S064X6S, S064X7A, S064X8A, S064X9A, S064X9D, S064X9S, S065X0A, S065X0D, S065X0S, S065X1A, S065X1D, S065X1S, S065X2A, S065X2D, S065X2S, S065X3A, S065X3D, S065X3S, S065X4A, S065X4D, S065X4S, S065X5A, S065X5D, S065X5S, S065X6A, S065X6D, S065X6S, S065X7A, S065X8A, S065X9A, S065X9D, S065X9S, S066X0A, S066X0D, S066X0S, S066X1A, S066X1D, S066X1S, S066X2A, S066X2D, S066X2S, S066X3A, S066X3D, S066X3S, S066X4A, S066X4D, S066X4S, S066X5A, S066X5D, S066X5S, S066X6A, S066X6D, S066X6S, S066X7A, S066X8A, S066X9A, S066X9D, S066X9S, I6000, I6001, I6002, I6010, I6011, I6012, I602, I6030, I6031, I6032, I604, I6050, I6051, I6052, I606, I607, I608, I609, I610, I611, I612, I613, I614, I615, I616, I618, I619, I6200, I6201, I6202, I6203, I621, I629, S06340A, S06340D, S06340S, S06341A, S06341D, S06341S, S06342A, S06342D, S06342S, S06343A, S06343D, S06343S, S06344A, S06344D, S06344S, S06345A, S06345D, S06345S, S06346A, S06346D, S06346S, S06347A, S06348A, S06349A, S06349D, S06349S, S0634AA, S0634AD, S0634AS, S06350A, S06350D, S06350S, S06351A, S06351D, S06351S, S06352A, S06352D, S06352S, S06353A, S06353D, S06353S, S06354A, S06354D, S06354S, S06355A, S06355D, S06355S, S06356A, S06356D, S06356S, S06357A, S06358A, S06359A, S06359D, S06359S, S0635AA, S0635AD, S0635AS, S06360A, S06360D, S06360S, S06361A, S06361D, S06361S, S06362A, S06362D, S06362S, S06363A, S06363D, S06363S, S06364A, S06364D, S06364S, S06365A, S06365D, S06365S, S06366A, S06366D, S06366S, S06367A, S06368A, S06369A, S06369D, S06369S, S0636AA, S0636AD, S0636AS, S06370A, S06370D, S06370S, S06371A, S06371D, S06371S, S06372A, S06372D, S06372S, S06373A, S06373D, S06373S, S06374A, S06374D, S06374S, S06375A, S06375D, S06375S, S06376A, S06376D, S06376S, S06377A, S06378A, S06379A, S06379D, S06379S, S0637AA, S0637AD, S0637AS, S06380A, S06380D, S06380S, S06381A, S06381D, S06381S, S06382A, S06382D, S06382S, S06383A, S06383D, S06383S, S06384A, S06384D, S06384S, S06385A, S06385D, S06385S, S06386A, S06386D, S06386S, S06387A, S06388A, S06389A, S06389D, S06389S, S0638AA, S0638AD, S0638AS, S064XAA, S064XAD, S064XAS, S065XAA, S065XAD, S065XAS, S066XAA, S066XAD, S066XAS |
| Non-ICH Critical Organ Bleed | H05231, H05232, H05233, H05239, H31301, H31302, H31303, H31309, H31311, H31312, H31313, H31319, H31411, H31412, H31413, H31419, H4310, H4311, H4312, H4313, H47021, H47022, H47023, H47029, I230, I312, K661, M2500, M25011, M25012, M25019, M25021, M25022, M25029, M25031, M25032, M25039, M25041, M25042, M25049, M25051, M25052, M25059, M25061, M25062, M25069, M25071, M25072, M25073, M25074, M25075, M25076, M2508, M7981, S2600XA, S2600XD, S2600XS, S2601XA, S2601XD, S2601XS, S26020A, S26020D, S26020S, S26021A, S26021D, S26021S, S26022A, S26022D, S26022S, S2609XA, S2609XD, S2609XS, G9519, S3701, S37011, S37011A, S37011D, S37011S, S37012, S37012A, S37012D, S37012S, S37019, S37019A, S37019D, S37019S, S3702, S37021, S37021A, S37021D, S37021S, S37022, S37022A, S37022D, S37022S, S37029, S37029A, S37029D, S37029S, S3703, S37031, S37031A, S37031D, S37031S, S37032, S37032A, S37032D, S37032S, S37039, S37039A, S37039D, S37039S, S3704, S37041, S37041A, S37041D, S37041S, S37042, S37042A, S37042D, S37042S, S37049, S37049A, S37049D, S37049S, S3705, S37051, S37051A, S37051D, S37051S, S37052, S37052A, S37052D, S37052S, S37059, S37059A, S37059D, S37059S, S3706, S37061, S37061A, S37061D, S37061S, S37062, S37062A, S37062D, S37062S, S37069, S37069A, S37069D, S37069S, H1130, H1131, H1132, H1133, H2100, H2101, H2102, H2103, H31321, H31322, H31323, H31329, H3560, H3561, H3562, H3563, H35731, H35732, H35733, H35739, H44811, H44812, H44813, H44819, J942, S271XXA, S271XXD, S271XXS |
| Gastrointestinal | I8501, I8511, K2211, K226, K250, K252, K254, K256, K260, K262, K264, K266, K270, K272, K274, K276, K280, K282, K284, K286, K2901, K2921, K2931, K2941, K2951, K2961, K2971, K2981, K2991, K31811, K3182, K50011, K50111, K50811, K50911, K51011, K51211, K51311, K51411, K51511, K51811, K51911, K5521, K5701, K5711, K5713, K5721, K5731, K5733, K5741, K5751, K5753, K5781, K5791, K5793, K625, K6381, K762, K920, K921, K922 |
| Other Bleed | D68311, D68312, D68318, D6832, D698, D699, R58, T792XXA, T792XXD, T792XXS, D62, T45511A, T45511D, T45511S, T45512A, T45512D, T45512S, T45513A, T45513D, T45513S, T45514A, T45514D, T45514S, T45515A, T45515D, T45515S, H61121, H61122, H61123, H61129, H9220, H9221, H9222, H9223, N020, N021, N022, N023, N024, N025, N026, N027, N028, N029, N421, N837, N897, N920, N921, N923, N924, N925, N930, N938, N939, N950, N99520, N99530, O717, R040, R041, R042, R0489, R049, R233, R310, R319 |
| **Comorbidities** | **ICD-10 Codes** |
| Atrial Fibrillation | I48, I480, I481, I4811, I4819, I482, I4820, I4821, I483, I484, I489, I4891, I4992 |
| Ischemic Heart Disease | I200, I201, I202, I208, I240, I241, I248, I2510, I25110, I25111, I25112, I25118, I25119, I253, I2541, I2542, I255, I256, I25700, I25701, I25702, I25708, I25710, I25711, I25712, I25718, I25719, I25720, I25721, I25722, I25728, I25729, I25730, I25731, I25732, I25738, I25739, I25750, I25751, I25752, I25758, I25759, I25760, I25761, I25762, I25768, I25769, I25790, I25791, I25792, I25798, I25799, I25810, I25811, I25812, I2582, I2583, I2584, I2589, I259 |
| Diabetes | E0821, E0822, E0829, E08311, E08319, E08321, E083211, E083212, E083213, E083219, E08329, E083291, E083292, E083293, E083299, E08331, E083311, E083312, E083313, E083319, E08339, E083391, E083392, E083393, E083399, E08341, E083411, E083412, E083413, E083419, E08349, E083491, E083492, E083493, E083499, E08351, E083511, E083512, E083513, E083519, E083521, E083522, E083523, E083529, E083531, E083532, E083533, E083539, E083541, E083542, E083543, E083549, E083551, E083552, E083553, E083559, E08359, E083591, E083592, E083593, E083599, E0836, E0837X1, E0837X2, E0837X3, E0837X9, E0839, E0840, E0841, E0842, E0843, E0844, E0849, E0851, E0852, E0859, E08610, E08618, E08620, E08621, E08622, E08628, E08630, E08638, E08641, E08649, E0865, E0869, E088, E0921, E0922, E0929, E09311, E09319, E09321, E093211, E093212, E093213, E093219, E09329, E093291, E093292, E093293, E093299, E09331, E093311, E093312, E093313, E093319, E09339, E093391, E093392, E093393, E093399, E09341, E093411, E093412, E093413, E093419, E09349, E093491, E093492, E093493, E093499, E09351, E093511, E093512, E093513, E093519, E093521, E093522, E093523, E093529, E093531, E093532, E093533, E093539, E093541, E093542, E093543, E093549, E093551, E093552, E093553, E093559, E09359, E093591, E093592, E093593, E093599, E0936, E0937X1, E0937X2, E0937X3, E0937X9, E0939, E0940, E0941, E0942, E0943, E0944, E0949, E0951, E0952, E0959, E09610, E09618, E09620, E09621, E09622, E09628, E09630, E09638, E09641, E09649, E0965, E0969, E098, E1021, E1022, E1029, E10311, E10319, E10321, E103211, E103212, E103213, E103219, E10329, E103291, E103292, E103293, E103299, E10331, E103311, E103312, E103313, E103319, E10339, E103391, E103392, E103393, E103399, E10341, E103411, E103412, E103413, E103419, E10349, E103491, E103492, E103493, E103499, E10351, E103511, E103512, E103513, E103519, E103521, E103522, E103523, E103529, E103531, E103532, E103533, E103539, E103541, E103542, E103543, E103549, E103551, E103552, E103553, E103559, E10359, E103591, E103592, E103593, E103599, E1036, E1037X1, E1037X2, E1037X3, E1037X9, E1039, E1040, E1041, E1042, E1043, E1044, E1049, E1051, E1052, E1059, E10610, E10618, E10620, E10621, E10622, E10628, E10630, E10638, E10641, E10649, E1065, E1069, E108, E1121, E1122, E1129, E11311, E11319, E11321, E113211, E113212, E113213, E113219, E11329, E113291, E113292, E113293, E113299, E11331, E113311, E113312, E113313, E113319, E11339, E113391, E113392, E113393, E113399, E11341, E113411, E113412, E113413, E113419, E11349, E113491, E113492, E113493, E113499, E11351, E113511, E113512, E113513, E113519, E113521, E113522, E113523, E113529, E113531, E113532, E113533, E113539, E113541, E113542, E113543, E113549, E113551, E113552, E113553, E113559, E11359, E113591, E113592, E113593, E113599, E1136, E1137X1, E1137X2, E1137X3, E1137X9, E1139, E1140, E1141, E1142, E1143, E1144, E1149, E1151, E1152, E1159, E11610, E11618, E11620, E11621, E11622, E11628, E11630, E11638, E11641, E11649, E1165, E1169, E118, E1321, E1322, E1329, E13311, E13319, E13321, E133211, E133212, E133213, E133219, E13329, E133291, E133292, E133293, E133299, E13331, E133311, E133312, E133313, E133319, E13339, E133391, E133392, E133393, E133399, E13341, E133411, E133412, E133413, E133419, E13349, E133491, E133492, E133493, E133499, E13351, E133511, E133512, E133513, E133519, E133521, E133522, E133523, E133529, E133531, E133532, E133533, E133539, E133541, E133542, E133543, E133549, E133551, E133552, E133553, E133559, E13359, E133591, E133592, E133593, E133599, E1336, E1337X1, E1337X2, E1337X3, E1337X9, E1339, E1340, E1341, E1342, E1343, E1344, E1349, E1351, E1352, E1359, E13610, E13618, E13620, E13621, E13622, E13628, E13630, E13638, E13641, E13649, E1365, E1369, E138E0800, E0801, E0810, E0811, E089, E0900, E0901, E0910, E0911, E099, E1010, E1011, E109, E1100, E1101, E1110, E1111, E119, E1300, E1301, E1310, E1311, E139, O24011, O24012, O24013, O24019, O2402, O2403, O24111, O24112, O24113, O24119, O2412, O2413, O24311, O24312, O24313, O24319, O2432, O2433, O24410, O24414, O24415, O24419, O24420, O24424, O24425, O24429, O24430, O24434, O24435, O24439, O24811, O24812, O24813, O24819, O2482, O2483, O24911, O24912, O24913, O24919, O2492, O2493 |
| Hypertension | I10, I160, I169, I1A0, O10011, O10012, O10013, O10019, O1002, O1003 H35031, H35032, H35033, H35039, I119, I129, I1310, I150, I151, I152, I158, I159, I161, I674, O10111, O10112, O10113, O10119, O1012, O1013, O10211, O10212, O10213, O10219, O1022, O1023, O10311, O10312, O10313, O10319, O1032, O1033, O10411, O10412, O10413, O10419, O1042, O1043, O10911, O10912, O10913, O10919, O1092, O1093, O111, O112, O113, O114, O115, O119, O161, O162, O163, O164, O165, O169 |
| History of Stroke | Z8673, I6930 |
| History of Venous Thromboembolism | Z8671, Z86711, Z86718, Z8672, I2782, I825, I8250, I82501, I82502, I82503, I82509, I8251, I82511, I82512, I82513, I82519, I8252, I82521, I82522, I82523, I82529, I8253, I82531, I82532, I82533, I82539, I8254, I82541, I82542, I82543, I82549, I8255, I82551, I82552, I82553, I82559, I8256, I82561, I82562, I82563, I82569, I8259, I82591, I82592, I82593, I82599, I825Y, I825Y1, I825Y2, I825Y3, I825Y9, I825Z, I825Z1, I825Z2, I825Z3, I825Z9 |
| Obesity | E6601, E6609, E661, E662, E668, E669, O99210, O99211, O99212, O99213, O99214, O99215, R939, Z6830, Z6831, Z6832, Z6833, Z6834, Z6835, Z6836, Z6837, Z6838, Z6839, Z6841, Z6842, Z6843, Z6844, Z6845, Z6854 |
| Chronic Kidney Disease | A1811, A5275, B520, E0821, E0822, E0829, E0921, E0922, E0929, E1021, E1022, E1029, E1121, E1122, E1129, E1321, E1322, E1329, I120, I129, I130, I1310, I1311, I132, K767, M1030, M10311, M10312, M10319, M10321, M10322, M10329, M10331, M10332, M10339, M10341, M10342, M10349, M10351, M10352, M10359, M10361, M10362, M10369, M10371, M10372, M10379, M1038, M1039, M3214, M3215, M3504, M350A, N010, N011, N012, N013, N014, N015, N016, N017, N018, N019, N01A, N020, N021, N022, N023, N024, N025, N026, N027, N028, N029, N02A, N030, N031, N032, N033, N034, N035, N036, N037, N038, N039, N03A, N040, N041, N042, N043, N044, N045, N046, N047, N048, N049, N04A, N050, N051, N052, N053, N054, N055, N056, N057, N058, N059, N05A, N060, N061, N062, N063, N064, N065, N066, N067, N068, N069, N06A, N070, N071, N072, N073, N074, N075, N076, N077, N078, N079, N07A, N08, N140, N141, N1411, N1419, N142, N143, N144, N150, N158, N159, N16, N181, N182, N183, N1830, N1831, N1832, N184, N185, N186, N189, N251, N2589, N259, N261, N269, N990, Q6102, Q6111, Q6119, Q612, Q613, Q614, Q615, Q618 |
| Heart Failure | I0981, I110, I130, I132, I501, I5020, I5021, I5022, I5023, I5030, I5031, I5032, I5033, I5040, I5041, I5042, I5043, I50810, I50811, I50812, I50813, I50814, I5082, I5083, I5084, I5089, I509 |
| Cancer | C9010, C9011, C9012, C9100, C9101, C9102, C9110, C9111, C9112, C9130, C9131, C9132, C9140, C9141, C9142, C9150, C9151, C9152, C9160, C9161, C9162, C9190, C9191, C9192, C91A0, C91A1, C91A2, C91Z0, C91Z1, C91Z2, C9200, C9201, C9202, C9210, C9211, C9212, C9220, C9221, C9222, C9230, C9231, C9232, C9240, C9241, C9242, C9250, C9251, C9252, C9260, C9261, C9262, C9290, C9291, C9292, C92A0, C92A1, C92A2, C92Z0, C92Z1, C92Z2, C9300, C9301, C9302, C9310, C9311, C9312, C9330, C9331, C9332, C9390, C9391, C9392, C93Z0, C93Z1, C93Z2, C9400, C9401, C9402, C9420, C9421, C9422, C9430, C9431, C9432, C9440, C9441, C9442, C946, C9480, C9481, C9482, C9500, C9501, C9502, C9510, C9511, C9512, C9590, C9591, C9592, C8100, C8101, C8102, C8103, C8104, C8105, C8106, C8107, C8108, C8109, C8110, C8111, C8112, C8113, C8114, C8115, C8116, C8117, C8118, C8119, C8120, C8121, C8122, C8123, C8124, C8125, C8126, C8127, C8128, C8129, C8130, C8131, C8132, C8133, C8134, C8135, C8136, C8137, C8138, C8139, C8140, C8141, C8142, C8143, C8144, C8145, C8146, C8147, C8148, C8149, C8170, C8171, C8172, C8173, C8174, C8175, C8176, C8177, C8178, C8179, C8190, C8191, C8192, C8193, C8194, C8195, C8196, C8197, C8198, C8199, C8200, C8201, C8202, C8203, C8204, C8205, C8206, C8207, C8208, C8209, C8210, C8211, C8212, C8213, C8214, C8215, C8216, C8217, C8218, C8219, C8220, C8221, C8222, C8223, C8224, C8225, C8226, C8227, C8228, C8229, C8230, C8231, C8232, C8233, C8234, C8235, C8236, C8237, C8238, C8239, C8240, C8241, C8242, C8243, C8244, C8245, C8246, C8247, C8248, C8249, C8250, C8251, C8252, C8253, C8254, C8255, C8256, C8257, C8258, C8259, C8260, C8261, C8262, C8263, C8264, C8265, C8266, C8267, C8268, C8269, C8280, C8281, C8282, C8283, C8284, C8285, C8286, C8287, C8288, C8289, C8290, C8291, C8292, C8293, C8294, C8295, C8296, C8297, C8298, C8299, C8300, C8301, C8302, C8303, C8304, C8305, C8306, C8307, C8308, C8309, C8310, C8311, C8312, C8313, C8314, C8315, C8316, C8317, C8318, C8319, C8330, C8331, C8332, C8333, C8334, C8335, C8336, C8337, C8338, C8339, C8350, C8351, C8352, C8353, C8354, C8355, C8356, C8357, C8358, C8359, C8370, C8371, C8372, C8373, C8374, C8375, C8376, C8377, C8378, C8379, C8380, C8381, C8382, C8383, C8384, C8385, C8386, C8387, C8388, C8389, C8390, C8391, C8392, C8393, C8394, C8395, C8396, C8397, C8398, C8399, C8400, C8401, C8402, C8403, C8404, C8405, C8406, C8407, C8408, C8409, C8410, C8411, C8412, C8413, C8414, C8415, C8416, C8417, C8418, C8419, C8440, C8441, C8442, C8443, C8444, C8445, C8446, C8447, C8448, C8449, C8460, C8461, C8462, C8463, C8464, C8465, C8466, C8467, C8468, C8469, C8470, C8471, C8472, C8473, C8474, C8475, C8476, C8477, C8478, C8479, C847A, C8490, C8491, C8492, C8493, C8494, C8495, C8496, C8497, C8498, C8499, C84A0, C84A1, C84A2, C84A3, C84A4, C84A5, C84A6, C84A7, C84A8, C84A9, C84Z0, C84Z1, C84Z2, C84Z3, C84Z4, C84Z5, C84Z6, C84Z7, C84Z8, C84Z9, C8510, C8511, C8512, C8513, C8514, C8515, C8516, C8517, C8518, C8519, C8520, C8521, C8522, C8523, C8524, C8525, C8526, C8527, C8528, C8529, C8580, C8581, C8582, C8583, C8584, C8585, C8586, C8587, C8588, C8589, C8590, C8591, C8592, C8593, C8594, C8595, C8596, C8597, C8598, C8599, C860, C861, C862, C863, C864, C865, C866, C880, C882, C883, C884, C888, C889, C9000, C9001, C9002, C9020, C9021, C9022, C9030, C9031, C9032, C960, C962, C9620, C9621, C9622, C9629, C964, C969, C96A, C96Z, D47Z9, C770, C771, C772, C773, C774, C775, C778, C779, C7800, C7801, C7802, C781, C782, C7830, C7839, C784, C785, C786, C787, C7880, C7889, C7900, C7901, C7902, C7910, C7911, C7919, C792, C7931, C7932, C7940, C7949, C7951, C7952, C7960, C7961, C7962, C7963, C7970, C7971, C7972, C7981, C7982, C7989, C799, C7B00, C7B01, C7B02, C7B03, C7B04, C7B09, C7B1, C7B8, C800, D0000, D0001, D0002, D0003, D0004, D0005, D0006, D0007, D0008, D001, D002, D010, D011, D012, D013, D0140, D0149, D015, D017, D019, D020, D021, D0220, D0221, D0222, D023, D024, D030, D0310, D0311, D03111, D03112, D0312, D03121, D03122, D0320, D0321, D0322, D0330, D0339, D034, D0351, D0352, D0359, D0360, D0361, D0362, D0370, D0371, D0372, D038, D039, D040, D0410, D0411, D04111, D04112, D0412, D04121, D04122, D0420, D0421, D0422, D0430, D0439, D044, D045, D0460, D0461, D0462, D0470, D0471, D0472, D048, D049, D0500, D0501, D0502, D0510, D0511, D0512, D0580, D0581, D0582, D0590, D0591, D0592, D060, D061, D067, D069, D070, D071, D072, D0730, D0739, D074, D075, D0760, D0761, D0769, D090, D0910, D0919, D0920, D0921, D0922, D093, D098, D099, C000, C001, C002, C003, C004, C005, C006, C008, C009, C01, C020, C021, C022, C023, C024, C028, C029, C030, C031, C039, C040, C041, C048, C049, C050, C051, C052, C058, C059, C060, C061, C062, C0680, C0689, C069, C07, C080, C081, C089, C090, C091, C098, C099, C100, C101, C102, C103, C104, C108, C109, C110, C111, C112, C113, C118, C119, C12, C130, C131, C132, C138, C139, C140, C142, C148, C153, C154, C155, C158, C159, C160, C161, C162, C163, C164, C165, C166, C168, C169, C170, C171, C172, C173, C178, C179, C180, C181, C182, C183, C184, C185, C186, C187, C188, C189, C19, C20, C210, C211, C212, C218, C220, C221, C222, C223, C224, C227, C228, C229, C23, C240, C241, C248, C249, C250, C251, C252, C253, C254, C257, C258, C259, C260, C261, C269, C300, C301, C310, C311, C312, C313, C318, C319, C320, C321, C322, C323, C328, C329, C33, C3400, C3401, C3402, C3410, C3411, C3412, C342, C3430, C3431, C3432, C3480, C3481, C3482, C3490, C3491, C3492, C37, C380, C381, C382, C383, C384, C388, C390, C399, C4000, C4001, C4002, C4010, C4011, C4012, C4020, C4021, C4022, C4030, C4031, C4032, C4080, C4081, C4082, C4090, C4091, C4092, C410, C411, C412, C413, C414, C419, C430, C4310, C4311, C43111, C43112, C4312, C43121, C43122, C4320, C4321, C4322, C4330, C4331, C4339, C434, C4351, C4352, C4359, C4360, C4361, C4362, C4370, C4371, C4372, C438, C439, C4400, C4409, C44101, C44102, C441021, C441022, C44109, C441091, C441092, C44131, C441321, C441322, C441391, C441392, C44191, C44192, C441921, C441922, C44199, C441991, C441992, C44201, C44202, C44209, C44291, C44292, C44299, C44300, C44301, C44309, C44390, C44391, C44399, C4440, C4449, C44500, C44501, C44509, C44590, C44591, C44599, C44601, C44602, C44609, C44691, C44692, C44699, C44701, C44702, C44709, C44791, C44792, C44799, C4480, C4489, C4490, C4499, C450, C451, C452, C457, C459, C460, C461, C462, C463, C464, C4650, C4651, C4652, C467, C469, C470, C4710, C4711, C4712, C4720, C4721, C4722, C473, C474, C475, C476, C478, C479, C480, C481, C482, C488, C490, C4910, C4911, C4912, C4920, C4921, C4922, C493, C494, C495, C496, C498, C499, C49A0, C49A1, C49A2, C49A3, C49A4, C49A5, C49A9, C4A0, C4A10, C4A11, C4A111, C4A112, C4A12, C4A121, C4A122, C4A20, C4A21, C4A22, C4A30, C4A31, C4A39, C4A4, C4A51, C4A52, C4A59, C4A60, C4A61, C4A62, C4A70, C4A71, C4A72, C4A8, C4A9, C50011, C50012, C50019, C50021, C50022, C50029, C50111, C50112, C50119, C50121, C50122, C50129, C50211, C50212, C50219, C50221, C50222, C50229, C50311, C50312, C50319, C50321, C50322, C50329, C50411, C50412, C50419, C50421, C50422, C50429, C50511, C50512, C50519, C50521, C50522, C50529, C50611, C50612, C50619, C50621, C50622, C50629, C50811, C50812, C50819, C50821, C50822, C50829, C50911, C50912, C50919, C50921, C50922, C50929, C510, C511, C512, C518, C519, C52, C530, C531, C538, C539, C540, C541, C542, C543, C548, C549, C55, C561, C562, C563, C569, C5700, C5701, C5702, C5710, C5711, C5712, C5720, C5721, C5722, C573, C574, C577, C578, C579, C58, C600, C601, C602, C608, C609, C61, C6200, C6201, C6202, C6210, C6211, C6212, C6290, C6291, C6292, C6300, C6301, C6302, C6310, C6311, C6312, C632, C637, C638, C639, C641, C642, C649, C651, C652, C659, C661, C662, C669, C670, C671, C672, C673, C674, C675, C676, C677, C678, C679, C680, C681, C688, C689, C6900, C6901, C6902, C6910, C6911, C6912, C6920, C6921, C6922, C6930, C6931, C6932, C6940, C6941, C6942, C6950, C6951, C6952, C6960, C6961, C6962, C6980, C6981, C6982, C6990, C6991, C6992, C700, C701, C709, C710, C711, C712, C713, C714, C715, C716, C717, C718, C719, C720, C721, C7220, C7221, C7222, C7230, C7231, C7232, C7240, C7241, C7242, C7250, C7259, C729, C73, C7400, C7401, C7402, C7410, C7411, C7412, C7490, C7491, C7492, C750, C751, C752, C753, C754, C755, C758, C759, C760, C761, C762, C763, C7640, C7641, C7642, C7650, C7651, C7652, C768, C7A00, C7A010, C7A011, C7A012, C7A019, C7A020, C7A021, C7A022, C7A023, C7A024, C7A025, C7A026, C7A029, C7A090, C7A091, C7A092, C7A093, C7A094, C7A095, C7A096, C7A098, C7A1, C7A8, D469, E3121, E3122, E3123 |

**SUPPLEMENTAL REFERENCES**

1. Shehab N, Ziemba R, Campbell KN, Geller AI, Moro RN, Gage BF, Budnitz DS, Yang TH. Assessment of ICD-10-CM code assignment validity for case finding of outpatient anticoagulant-related bleeding among Medicare beneficiaries. Pharmacoepidemiol Drug Saf. 2019;28:951-964.
2. Joos C, Lawrence K, Jones AE, Johnson SA, Witt DM. Accuracy of ICD-10 codes for identifying hospitalizations for acute anticoagulation therapy-related bleeding events. Thromb Res. 2019;181:71-76.
3. Yokoyama S, Tanaka Y, Nakagita K, Hosomi K, Takada M. Bleeding Risk of Warfarin and Direct Oral Anticoagulants in Younger Population: A Historical Cohort Study Using a Japanese Claims Database. Int J Med Sci. 2018;15:1686-1693.
